# Supplementary material for: A pentose, as a cytosine nucleobase modification in Shewanella phage Thanatos genomic DNA, mediates enhanced resistance toward host restriction systems
Source: Appl Environ Microbiol. 2025 Dec 29;92(1):e01333-25. doi: 10.1128/aem.01333-25 (PMC12838424; doi:10.1128/aem.01333-25)
Supplement: Supplemental material — Tables S1 to S4; Fig. S1 to S6. [file aem.01333-25-s0001.pdf]

A pentose as a cytosine nucleobase modification in *Shewanella* phage Thanatos genomic DNA mediates enhanced resistance towards host restriction systems

David Brandt[1], Anja K. Dörrich[2], Marcus Persicke[1], Alina Kemmler[2], Tabea Leonhard[2], Markus Haak[1], Sophia Nölting[1], Matthias Ruwe[1], Nicole Schmid[2], Kai M. Thormann[2*], and Jörn Kalinowski[1,*]

[1] Center for Biotechnology (CeBiTec), Bielefeld University, Bielefeld, Germany

[2] Department of Microbiology and Molecular Biology, Justus Liebig University Giessen, Giessen, Germany

Supplementary Material

Contains: Supplementary Tables S1 – S4
Supplementary Figures S1 – S6

Supplementary Table S1: Top 20 structural homology hits from DALI against PDB25 for the predicted protein structure of TH1_063.

#No	Chain	Z	rmsd	lali	nres	%id	PDB Description
1	6kih-A	18	3,4	269	400	8	TLL1590 PROTEIN;
2	4xsu-A	17,4	3,1	256	368	9	ALR3699 PROTEIN;
3	6n1x-A	17,4	3	255	377	8	GLYCOSYLTRANSFERASE;
4	2iv7-A	15,4	3,3	249	370	10	LIPOPOLYSACCHARIDE CORE BIOSYNTHESIS PROTEIN
5	6gng-B	15,3	3,4	260	526	8	GRANULE-BOUND STARCH SYNTHASE;
6	4xyw-A	15,2	3,5	241	324	10	O-ANTIGEN BIOSYNTHESIS GLYCOSYLTRANSFERASE WBNH;
7	2iuy-A	15,2	3,1	236	340	12	GLYCOSYLTRANSFERASE;
8	5zer-A	14,8	3,2	244	354	10	UDP-GLUCOSE:TETRAHYDROBIOPTERIN GLUCOSYLTRANSFERA
9	6eji-A	14,7	3,1	239	360	11	WLAC PROTEIN;
10	2bis-A	14,6	3,8	257	440	12	GLGA GLYCOGEN SYNTHASE;
11	3oka-A	14,3	3,8	250	378	8	GDP-MANNOSE-DEPENDENT ALPHA-(1-6)-PHOSPHATIDYLINO
12	7mi0-A	14,3	3,7	244	379	13	GLYCOSYLTRANSFERASE;
13	4wac-A	14,2	3,6	252	498	11	GLYCOSYL TRANSFERASE, GROUP 1 FAMILY PROTEIN;
14	4pqg-A	13,8	3,2	241	506	10	GLYCOSYLTRANSFERASE GTF1;
15	4n9w-A	13,8	3,3	225	360	8	GDP-MANNOSE-DEPENDENT ALPHA-(1-2)-PHOSPHATIDYLINO
16	5uof-A	13,8	3,7	252	471	10	ALPHA,ALPHA-TREHALOSE-PHOSPHATE SYNTHASE (UDP-FOR
17	5tmb-A	13,4	4,2	250	451	8	GLYCOSYLTRANSFERASE, OS79;
18	4fkz-A	13,4	4,3	258	384	10	UDP-N-ACETYLGLUCOSAMINE 2-EPIMERASE;
19	1xv5-A	13,2	3,5	239	401	8	DNA ALPHA-GLUCOSYLTRANSFERASE;
20	2hy7-A	13,2	4,2	238	373	5	GLUCURONOSYLTRANSFERASE GUMK;

Supplementary Table S2: Bacterial strains used in this study

Strain	Genotype	Purpose	Reference
Escherichia coli			
DH5α-λpir	φ80d*lacZ* ΔM15 Δ(*lacZYA-argF*)U169 *recA*$_1$ *hsdR17 deoR thi-l supE44 gyrA96 relA*$_1$/*λpir*	regular cloning strain	Miller & Mekalanos, 1988 (doi: 10.1128/jb.170.6.2575-2583.1988)
WM3064	*thrB1004 pro thi rpsL hsdS lacZ* ΔM15 RP4-1360 Δ(*araBAD*) 567Δ*dapA* 1341::[*erm pir*(wt)]	conjugation strain	W. Metcalf, University of Illinois, Urbana-Champaign
ER3413	*fhuA2::IS2, glnX44(AS), λ-, e14-, trp-31, dcm-6, yedZ3069::Tn10, hisG1, argG6, yhdJ11, rpsL104, Δdam-16::KanR, xyl-7, mtlA2, metB1, Δ(mcrC-mrr)114:IS10*	expression strain	E.A. Raleigh, Complete Genome Sequence of ER2796, a DNA Methyltransferase-Deficient Strain of Escherichia coli K-12. PLoS ONE 10(5):e0127446-.
BL21-Gold(DE3)	*ompT hsdS(rB – mB –) dcm+ Tetr gal λ(DE3) endA Hte*	expression strain	Agilent
Shewanella oneidensis MR-1			
S79	*S. oneidensis* MR-1 wild type		Venkateswaran et al, 1999 (doi: 10.1099/00207713-49-2-705)
S1419 (S6593)	MR-1 ΔLambdaSo ΔMuSo2 (deletion of active prophages)		Gödeke et al, 2011 (doi: 10.1038/ismej.2010.153)
S6733	S1419 + pCASCADE_RBS-Cas1,wtCas3 + pBAD33 (negative control)		this work
S6735	S1419 + pCASCADE_RBS-Cas1,wtCas3 + pCRISPR λ SO_2963		this work
S6737	S1419 + pCASCADE_RBS-Cas1,wtCas3 + pCRISPR λ SO_2975		this work
S6741	S1419 + pCASCADE_RBS-Cas1,wtCas3 + pCRISPR Thanatos TH1_010		this work
S6743	S1419 + pCASCADE_RBS-Cas1,wtCas3 + pCRISPR Thanatos TH1_20		this work
S6086	S1419 + pTS021 Ara (negative control)		this work
S6197	S1419 + pTS021 Ara gRNA SO_2963		this work
S6313	S1419 + pTS021 Ara gRNA SO_2953		this work
S6209	S1419 + pTS021 Ara gRNA TH1_062		this work
S6210	S1419 + pTS021 Ara gRNA TH1_126		this work
S9405	S1419 + pBAD33_TH1_60_frameshift pBBR1MCS-5_TH1_60		this work
S9566	S1419 + pBBR1MCS-5_TH1_60		this work

Supplementary Table S3: Plasmids used in this study

**Plasmid designation**	**Description**	Reference
pBBR-MCS5	broad-range cloning plasmid, GmR	Kovach et al, 1995 (doi: 10.1016/0378-1119(95)00584-1)
pBAD33	pACYC184/p15A; *araC*-P_{BAD}; CmR	Guzman et al, 1995 (doi: 10.1128/jb.177.14.4121-4130.1995)
pCas1	Expression vector of the *Shewanella putrefaciens* CN-32 I-Fv type CRISPR-Cas genes *cas7fv*, *cas5fv* and *cas6f*	Gleditzsch et al, 2016 (doi: 10.1093/nar/gkw469)
pCASCADE_RBS_Cas1	Cascade genes (*cas7fv cas5fv cas6f*) with a RBS placed in front of *cas7fv* and a FLAG tag added to *cas6f* from pCas1 placed under the control of the ArcA-P_{BAD} promoter cassette from BAD33 in pBBR-MCS5, GmR	this work
pCRISPR λ SO_2963	arabinose-inducible expression of guide RNA targeting LambdaSo gene SO_2963	this work
pCRISPR λ SO_2975	arabinose-inducible expression of guide RNA targeting LambdaSo gene SO_2975	this work
pTS021	ori-p15A, *lacPOZ*, *cas9*, P_{man}, P_{van}, KmR	Otte et al, 2020 (https://doi.org/10.1111/lam.13349)
pTS021-ara	ori-p15A, *lacPOZ*, *araC-P_{BAD}-cas9*, P_{man}, P_{van}, KmR	this work
pTS021-ara_TH1_062	gRNA targeting TH1_062 in pTS021-ara	this work
pTS021-ara_TH1_126	gRNA targeting TH1_126 in pTS021-ara	this work
pBAD24-TH126	Arabinose-inducible expression of phage protein TH1_126	this work
pBAD24-NHis-TH1-060	Arabinose-inducible expression of phage protein TH1_060	this work
pBAD24-NHis-TH1-063	Arabinose-inducible expression of phage protein TH1_063	this work
pBAD33_TH1_60_frame shift	Used for phage engineering	this work
pBBR1MCS-5_TH1_60	Complementation of Thanatos mutant missing a functional TH1_60 gene	this work

Supplementary Table S4: Oligonucleotides used in this study

Designation/Purpose	Sequence (5'→3')
Construction of pCASCADE	
pBAD33-AraC-FW	GTGGATCCCCCGGGCTGCAGGTTATGACAACTTGACGGCTAC
pBAD33-Pbad-OL-Cas1-RV	GCTAAAATCATCCATGCTAGCCCAAAAAAACGGGTATGG
RBS-3xFLAG-FW	CGGGGATCCTCTAGAGAGGAGGTGCATCATGGATTATAAAGATCATGATGGCGATTATAAAGATCATGATATTGATTATAAAGATGATGATGATAAATAAGGCATGCAAGCTTGGC
3xFLAG-RV	GCCAAGCTTGCATGCCTTATTTATCATCATCATCTTTATAATCAATATCATGATCTTTATAATCGCCATCATGATCTTTATAATCCATGATGCACCTCCTCTCTAGAGGATCCCCG
pBBR1-MCS5-check-FW	GCGCGTAATACGACTCACTATAGG
pBBR1-MCS5-check-RV	CCTCACTAAAGGGAACAAAAGCTGG
Cloning of targeting spacers into pBAD33	
λSO_2963-Spacer-FW	CGGGGATCCTCTAGAGGTTCACCGCCGCACAGGCGGCTTAGAAAAGAGGCATTTGTTAAGGGTAACTTTACTTTTGGTTCACCGCCGCACAGGCGGCTTAGAAAGGCATGCAAGCTTGGC
λSO_2963-Spacer-RV	GCCAAGCTTGCATGCCTTTCTAAGCCGCCTGTGCGGCGGTGAACCAAAAGTAAAGTTACCCTTAACAAATGCCTCTTTTCTAAGCCGCCTGTGCGGCGGTGAACCTCTAGAGGATCCCCG
pBAD33-check-FW	GCCGTCAATTGTCTGATTCG
pBAD33-check-RV	GTTTTATCAGACCGCTTCTGCG
λSO_2975-Spacer-FW	CGGGGATCCTCTAGAGGTTCACCGCCGCACAGGCGGCTTAGAAAGTACTAGCGGCGGCAGATTTATCGGCTGGAGCGTTCACCGCCGCACAGGCGGCTTAGAAAGGCATGCAAGCTTGGC
λSO_2975-Spacer-RV	GCCAAGCTTGCATGCCTTTCTAAGCCGCCTGTGCGGCGGTGAACGCTCCAGCCGATAAATCTGCCGCCGCTAGTACTTTCTAAGCCGCCTGTGCGGCGGTGAACCTCTAGAGGATCCCCG
TH_010 (0047)-Spacer-FW	CGGGGATCCTCTAGAGGTTCACCGCCGCACAGGCGGCTTAGAAAACGGTTATAATGCTACTAACATCGCTTCAGGTGTTCACCGCCGCACAGGCGGCTTAGAAAGGCATGCAAGCTTGGC
TH_010 (0047)-Spacer-RV	GCCAAGCTTGCATGCCTTTCTAAGCCGCCTGTGCGGCGGTGAACACCTGAAGCGATGTTAGTAGCATTATAACCGTTTTCTAAGCCGCCTGTGCGGCGGTGAACCTCTAGAGGATCCCCG
TH_020 (0057)-Spacer-FW	CGGGGATCCTCTAGAGGTTCACCGCCGCACAGGCGGCTTAGAAAAAGTTATGAATCTAATGCTATTTTAGTAGAGAGTTCACCGCCGCACAGGCGGCTTAGAAAGGCATGCAAGCTTGGC
TH_020 (0057)-Spacer-RV	GCCAAGCTTGCATGCCTTTCTAAGCCGCCTGTGCGGCGGTGAACTCTCTACTAAAATAGCATTAGATTCATAACTTTTTCTAAGCCGCCTGTGCGGCGGTGAACCTCTAGAGGATCCCCG
Construction of pTS021-ara and derivatives (Cas9)	
TL 149	CACTTCCCTGTTAAGTGTACTTATGACAACTTGACGGCTA
TL 177	ATGTCATGACATTGGTGTACACAGTAGAGAGTTGCGATAAA
TL 176	CCACCACTGATTTGAGCGTCAG
TL 163	CGTCAGATTTCGTGATGCTTGTC
RH001	AGCTTAGGCCCAGTCGAAAG

RH002	CAGCTAGGAGGTGACTGAAG
RH003	ACCGAGCGTTCTGAACAAATCC
gRNA TH_062 (T_00098)-f	TACGTAAAAAGAATAGTCGTCGTG
gRNA TH_062 (T_00098)-r	AAACCACGACGACTATTCTTTTTA
gRNA TH_126 (T_00161)-f	TACGCATGTAGAGCATTTCACTAG
gRNA TH_126 (T_00161)-r	AAACCTAGTGAAATGCTCTACATG
Cloning of inducible expression of TH1_126 into NcoI site of pBAD24	
pBAD_TH1-126_fw	GCTAGCAGGAGGAATTCACATGATTAATTATAAAGATGGTCTAAATGG
pBAD_TH1-126_rev	TCTAGAGGATCCCCGGGTACCTAATAGTTAGTTATCAACACTTCTAC
Cloning of inducible expression of TH1_060 and TH1_063 into NcoI site of pBAD24.	
pBAD_TH1-060_fwd	GGCTAGCAGGAGGAATTCACATGAAAAAAGTTGTTATTCTTGCTTCTGGTATG G
pBAD_TH1-060_rev	TCTAGAGGATCCCCGGGTACTCATAGTTTTTCCCATTGGTGGTCAATATGAC
pBAD_TH1-063_fwd	GGCTAGCAGGAGGAATTCACATGAATTTTATCGTCCCTATATTTTCAATGCGT
pBAD_TH1_063_rev	TCTAGAGGATCCCCGGGTACTCATGTTACCACCAAATCATTGATTAGAGGC
Addition of 6x N-terminal His-tags via primer overhangs	
pBAD_split_f_1	CGTTGCGCAAACTATTAACTG
pBAD_NHis_r_1	GTGGTGATGGTGATGATGCATGTGAATTCCTCCTGCTAGCC
pBAD_TH1-60_NHis_f_2	CATCATCACCATCACCACAAAAAAGTTGTTATTCTTGCTTCTGG
pBAD24_split_r_2	AGTTAATAGTTTGCGCAACG
pBAD24_TH1-63_NHis_f_2	CATCATCACCATCACCACAATTTTATCGTCCCTATATTTTC
Construction of Knockout TH1_60	
NES605 pBAD_TH1-60 frameshift 1 FWD	AATTCGAGCTCGGTACCCCATTTAGGTTTTCTTTAGTATCAGGC
NES606 pBAD_TH1-60 frameshift 1 REV	TAATCAATATCATGATCTTTATAATCGCCATCATGATCTTTATAATCGAAAAAAG TTGTTATTCTTGCTTC
NES607 pBAD_TH1-60 frameshift 2 FWD	ATTATAAAGATCATGATATTGATTATAAAGATGATGATGATAAATAAAATGTTC TCCGTTTAAAAATTG
NES608 pBAD_TH1-60 frameshift 2 REV	CGACTCTAGAGGATCCCCCTAATATTACTACCATCTGTGGGTTAA
Cloning of complementation plasmid	
NES658 pBBR_TH1_60 FWD	AGAACTAGTGGATCCCCCTCATAGTTTTTCCCATTGGTG
NES659 pBBR_TH1_60 REV	ATCGAATTCCTGCAGCCCAGGAGGGCAAATATGAAAAAAGTTGTTATTCTTGCT TC
Check for Knockout of TH1_60	
NES578 TH1_28_29 FLAG check REV	CAATATCATGATCTTTATAATCGCC
NES609_TH1_60 check REV	GAATCAAAGCATTACGACCTAG
AK118 TH1_60 KO check fw	GCTCTAGTTCAAGTTGCGATTAA
AK119 TH1_60 KO check rev	TCCAAGCTGTTATAGTATTATCGA

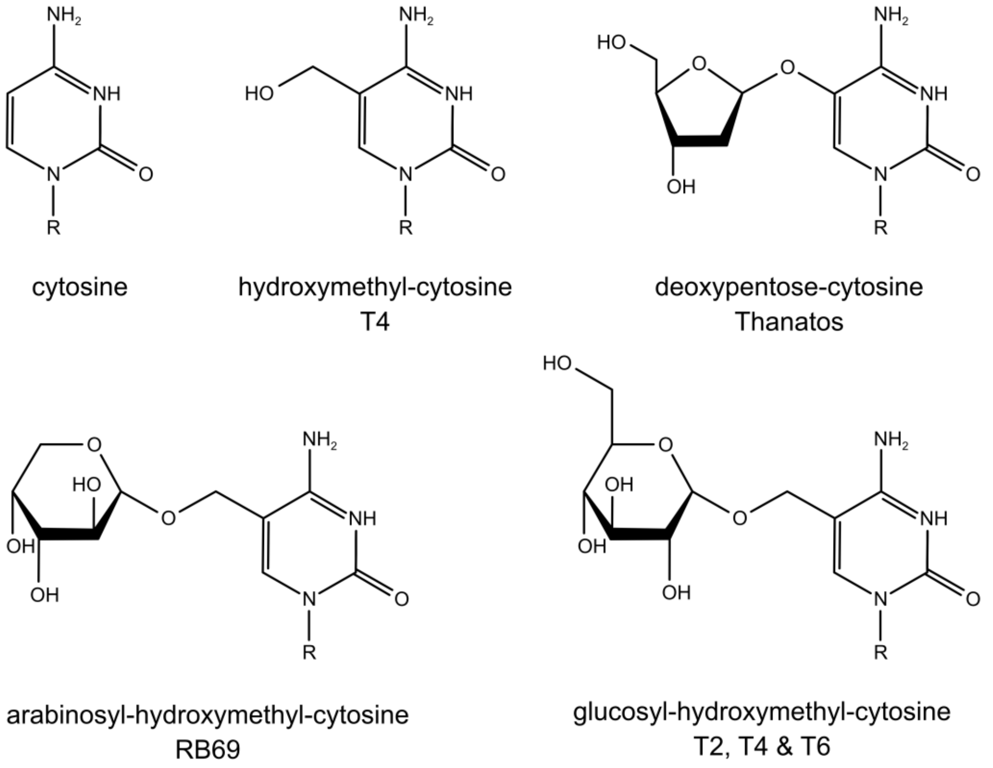

Supplementary Figure S1: Examples for known cytosine modification variants used by phages. The corresponding phage name are given below. In the upper right corner is the predicted modification of *Shewanella* phage Thanatos-1, whose identification is described in this study.

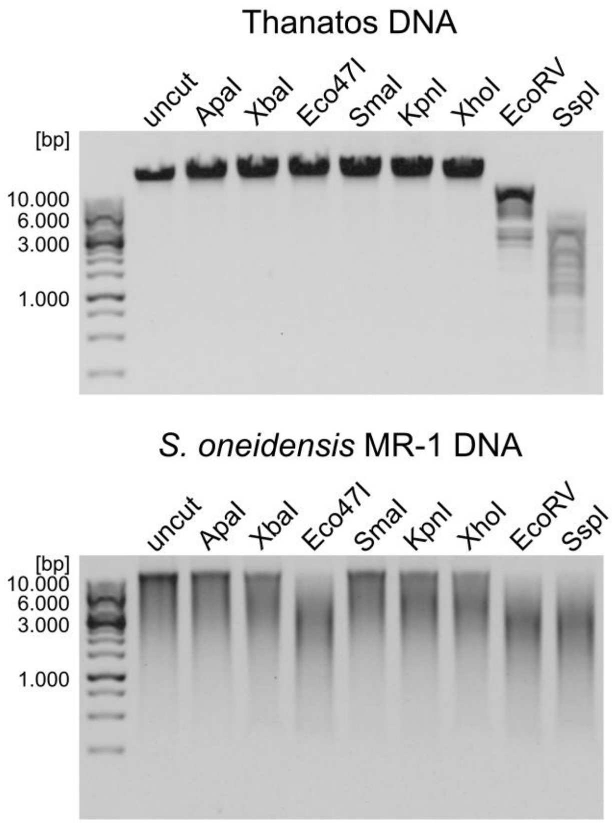

Supplementary Figure S2: Restriction of *S. oneidensis* MR-1 and *Shewanella* phage Thanatos-1 DNA by type II restriction enzymes. Shown is a separation of DNA on an 0.7 % (w/v) agarose gel after following 3 h of digestion at the appropriate temperatures. As indicated, the following enzymes were used: ApaI (GGGCCC; 12 predicted sites for Thanatos); XbaI (TCTAGA, 75 pred. sites); Eco47I (GGWCC; 96 predicted sites); SmaI (CCCGGG; 11 sites); KpnI (GGTACC; 8 sites); XhoI (CTCGAG; 4 sites); EcoRV (GATATC; 37 sites); SspI (AATAAT; 193 sites). Pronounced digestion of Thanatos-1 DNA only occurred when EcoRV or SspI was used; however, it is unclear if the digestion was complete.

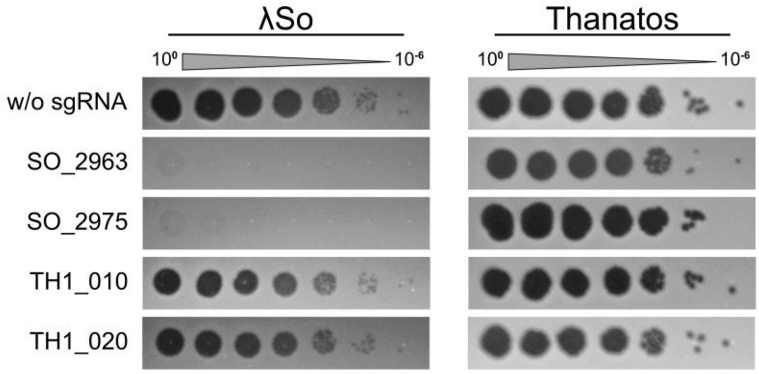

Supplementary Figure S3: Thanatos is resistant to Cas3 while LambdaSo DNA is cleaved. A spot assay of *S. oneidensis* MR-1 is shown, with cells either carrying the empty vector (w/o sgRNA) or a vector expressing an sgRNA against λSo (SO_2963, SO_2975) or Thanatos (TH1_010, TH1_020). The plasmid additionally carries the CRISPR-Cas I-fv system from *S. putrefaciens* CN32 (Cas3). While Thanatos is completely resistant to the Cas3 system, λSo is sensitive to the spacers against λSo DNA but resistant to the spacers against Thanatos. The experiment was performed in biological triplicates.

Characterization of an adenosine 6mA methyltransferase specifically active on NA*TC sequence motifs

To find out whether base composition upstream or downstream of the ATC trimer influences methylation frequency, we extracted all k-mers containing ATC and extended these both upstream and downstream with up to three occurrences of each base. All instances of the resulting set of k-mers of length 4-6 bp in the ER3413 genome sequence were analyzed with regard to modification frequency. It turned out that methylation signals in the overexpression strain at GATC sites are the strongest, with AATC, TATC and CATC following in decreasing order (Figure 4A). The same analysis conducted for the negative control shows that, firstly, there is no substantial methylation at NATC sites, and that, secondly, there seems to be a sequence context-based effect, with GATC and AATC sites possessing a higher perceived modification rate compared to the other tetramers (Figure 4B). Conclusively, it is highly likely that the higher modification rate at GATC and AATC sites in the overexpression strain results from a context-based bias of tombo's 6mA model, and in reality, there is no preference of any base upstream of the 5'-ATC-3' motif.

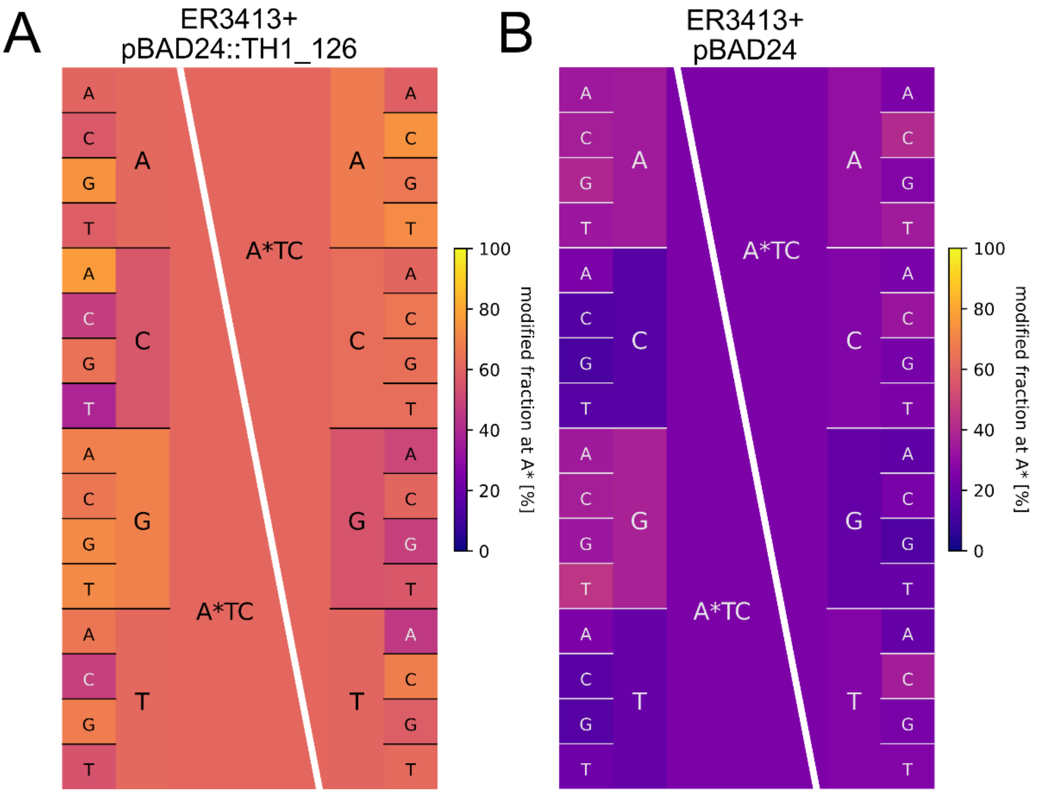

Supplementary Figure S4: Analysis of all ATC-containing sequences in the ER3413 sequence data with regard to the modified fraction of adenine in the ATC motif. Comparison of negative control (B) and methylase overexpression strain (A) shows little unspecific predicted modification in the negative control and a substantial predicted fraction of modified bases in the overexpression strain. The ATC core motif is extended upstream and downstream individually by all four bases and the color scale depicts the mean fraction of modified adenines in the respective k-mers as calculated by tombo v1.5 (20).

The *E. coli* MG1655 gDNA with a wild-type methylation profile, which includes dam methylation, was readily cleaved by DpnI (+) as visible by the decrease of DNA fragment lengths, whereas untreated DNA (-) retained its high molecular weight. This was to be expected, since DpnI is known to cleave methylated DNA at NATC sites and thereby proves the activity of the employed DpnI enzyme.

Integration of empty pBAD24 into ER3413 did not have any effect on the cleavage efficiency of its gDNA even upon the addition of DpnI (+), since ER3413 does not have methylated gDNA and the empty pBAD vector does not introduce any heterologous methylase.

The heterologous expression of TH1_126 via arabinose induction in ER3413, however, led to a shift of the DNA smear towards smaller fragments upon the addition of DpnI (+), providing proof for cleavage of ER3413 gDNA, thereby showing the activity of TH1_126 on *E. coli* DNA *in vivo* methylating NATC motifs.

Interestingly, although 5'-ATC-3' is part of the EcoRV recognition site (GATATC), the restriction assays of Thanatos-1 DNA showed substantial cleavage by EcoRV (Supplementary Figure S2).

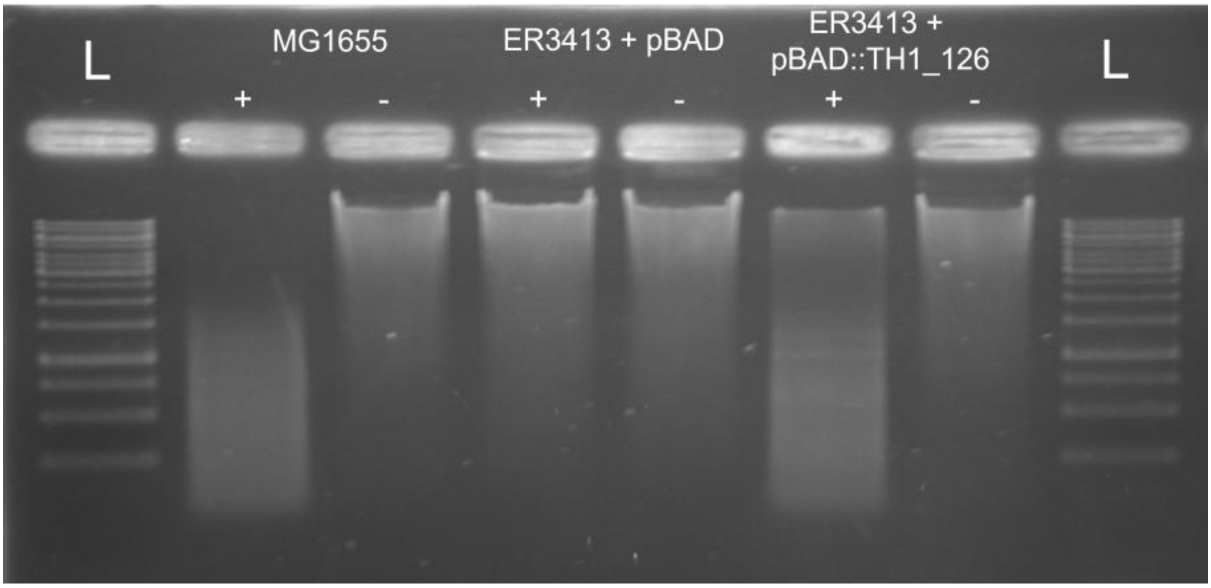

Supplementary Figure S5: *E. coli* MG1655 gDNA with a wild-type methylation profile, which includes dam methylation, was readily cleaved by DpnI. This is visible by the decrease of DNA fragment lengths, whereas untreated DNA retained its high molecular weight. Integration of empty pBAD24 into ER3413 did not have any effect on the cleavage efficiency of its gDNA. The heterologous expression of TH1_126 via arabinose induction in ER3413, however, led to a shift of the DNA smear towards smaller fragments, providing proof for cleavage of ER3413 gDNA, thereby showing the activity of TH1_126 on *E. coli* DNA *in vivo* methylating NATC motifs. Interestingly, although 5'-ATC-3' is part of the EcoRV recognition site (GATATC), the restriction assays of Thanatos-1 DNA showed substantial cleavage by EcoRV (Supplementary Figure 1).

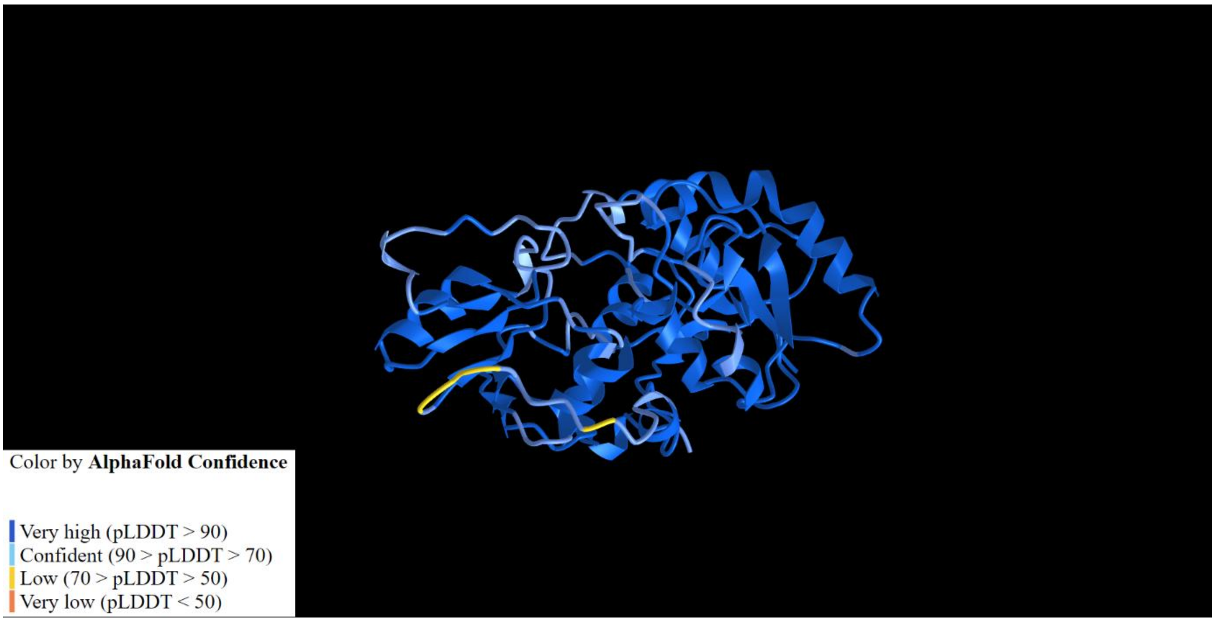

Supplementary Figure S6: Predicted protein structure of TH1_063 as determined by the ColabFold implementation of the AlphaFold algorithm. ColabFold outputs five structures ranked by the confidence emitted from the AlphaFold algorithm. This figure shows the top-ranked structure that was also used for DALI search.
